# Decision-making pathways of voluntary sexual partner disclosure among newly diagnosed people living with HIV: a qualitative study informed by the health belief model

**DOI:** 10.3389/fpubh.2026.1863157

**Published:** 2026-06-08

**Authors:** Yu Luo, Qing Yu

**Affiliations:** Department of Infectious Diseases, Chongqing Public Health Medical Center, Chongqing, China

**Keywords:** decision-making process, health belief model, newly diagnosed people living with HIV, qualitative research, sexual partner disclosure

## Abstract

**Objective:**

To explore the decision-making pathways and influencing factors of voluntary sexual partner disclosure among newly diagnosed people living with HIV, informed by the Health Belief Model.

**Methods:**

A descriptive qualitative design was adopted. Guided by the HBM, semi-structured in-depth interviews were conducted with 31 newly diagnosed people living with HIV between January and March 2026. Participants were purposively recruited from a tertiary infectious disease hospital in Chongqing, China, including both inpatient wards and outpatient clinics. Data collection and analysis were conducted concurrently and continued until thematic saturation was achieved. Data were analyzed using directed content analysis, while allowing for the retention of emerging themes beyond the original theoretical framework.

**Results:**

Six themes and 18 subthemes were identified: (1) emotional shock, (2) risk perception, (3) perceived benefits, (4) barriers, (5) disclosure pathways, and (6) outcomes and support. Participants commonly experienced significant emotional shock during the early stage after diagnosis. Perceived health risks to sexual partners and the expected benefits of disclosure served as important foundations for voluntary disclosure, whereas stigma-related concerns, perceived relationship risks, concerns about privacy control, and insufficient knowledge and communication confidence constituted major barriers. Through the dynamic balancing of these factors, participants mainly followed three pathways: direct disclosure, delayed disclosure after internal deliberation, and non-disclosure.

**Conclusion:**

Voluntary sexual partner disclosure among newly diagnosed people living with HIV is a dynamic decision-making process shaped by risk perception, benefit appraisal, and barrier evaluation. Disclosure support should be moved forward to the early post-diagnosis stage, with enhanced communication guidance, psychosocial support, and privacy protection provided alongside disease-related knowledge support, so as to promote more timely and appropriate partner disclosure.

## 1 Introduction

Human immunodeficiency virus (HIV) infection remains a major global public health issue. According to the Joint United Nations Programme on HIV/AIDS, approximately 40.8 million people were living with HIV worldwide in 2024, with about 1.3 million new infections, and a proportion of infected individuals still remained undiagnosed, indicating that HIV prevention and control continue to face substantial challenges (1). The World Health Organization has emphasized that HIV testing services should include not only testing itself, but also post-test counseling, linkage to treatment and care, and partner-related services. Among these, partner notification helps identify sexual partners who may have been exposed and facilitates timely testing and follow-up interventions (2). Therefore, optimizing partner services after diagnosis is important not only for individual linkage to care, but also for the prevention of secondary transmission and the improvement of public health service quality (3, 4).

For newly diagnosed people living with HIV (PLWH), voluntary disclosure to sexual partners is not merely a matter of information sharing, but a complex decision involving health responsibility, relationship negotiation, privacy management, and psychosocial adaptation (5). In the early stage after diagnosis, individuals often experience significant emotional shock, while also facing concerns about partner infection risk, relationship breakdown, privacy disclosure, and uncertainty about the future. As a result, whether, when, and how to disclose often becomes an important practical issue soon after diagnosis (6).

Previous studies have shown that rates of sexual partner disclosure among PLWH remain suboptimal and vary substantially across different types of partner relationships (7). Factors such as protecting partners’ health, fulfilling relationship responsibilities, obtaining support, and receiving advice from healthcare professionals may facilitate voluntary disclosure, whereas stigma and discrimination concerns, fear of relationship breakdown, worries about privacy disclosure, and communication difficulties may constitute important barriers (8, 9). However, existing studies have mainly focused on disclosure rates among the broader population of PLWH (10), associated factors (11–13), and disclosure status in some newly diagnosed groups (14, 15). Process-oriented research examining how newly diagnosed PLWH weigh these factors in the early post-diagnosis period, why they delay or refuse disclosure, and how their disclosure decisions evolve across changing contexts remains limited (15).

The Health Belief Model (HBM) provides an appropriate theoretical perspective for understanding this behavioral process. The HBM proposes that whether an individual adopts a health-related behavior is mainly influenced by perceived susceptibility, perceived severity, perceived benefits, perceived barriers, cues to action, and self-efficacy (16). For newly diagnosed PLWH, voluntary disclosure to sexual partners is essentially a health-related decision-making behavior and is therefore well suited to analysis within the HBM framework. Accordingly, this study, guided by the HBM, used a qualitative approach to explore the decision-making process and influencing factors of voluntary sexual partner disclosure among newly diagnosed PLWH, with the aim of informing the optimization of post-diagnosis HIV counseling, partner services, and stratified support strategies (16, 17).

## 2 Methods

### 2.1 Participants

Purposive sampling was used in this study. Newly diagnosed PLWH were recruited from the infectious disease wards and outpatient clinic of Chongqing Public Health Medical Center between January and March 2026. To enhance heterogeneity and validity of the study, participants were selected with consideration of variation in age, marital status, educational level, sexual orientation, partner type, and whether they had already undertaken voluntary disclosure to their sexual partners.

The inclusion criteria were as follows: (1) aged 18 years or older; (2) diagnosed within 6 months prior to study enrollment; (3) having had a sexual partner within the past 6 months; (4) having faced, currently facing, or having already completed the decision-making process regarding voluntary disclosure of HIV status to that partner; (5) able to communicate verbally or in writing with study staff (with or without assistance); (6) able to participate in the interview in Chinese; (7) providing informed consent and agreeing to participate voluntarily. The exclusion criteria were as follows: (1) any condition that, in the interviewer’s judgment, would preclude the participant from providing informed consent or completing the interview; and (2) acute clinical instability that made participation in a 30–60-min interview inappropriate or unsafe, as determined by medical records, clinical judgment, or patient report. For analysis, participants who withdrew during the interview or had incomplete audio recordings were excluded. A total of 31 participants were ultimately included. Data collection and analysis were conducted concurrently, and recruitment was discontinued when thematic saturation was reached.

### 2.2 Study design

This study adopted a descriptive qualitative design informed by the Health Belief Model (HBM). The HBM includes six core constructs: perceived susceptibility, perceived severity, perceived benefits, perceived barriers, cues to action, and self-efficacy and provides a useful framework for understanding health-related decision-making. In the present study, the HBM was used primarily as a theoretical guide to inform the development of the interview guide and the analytic process, including open coding followed by mapping emergent themes onto HBM constructs where relevant, while remaining open to themes outside the model, rather than as a rigid framework into which all participant experiences were forced. This was important because voluntary disclosure to sexual partners during the newly diagnosed stage is shaped not only by cognitive appraisal, but also by emotional reactions, interpersonal context, and evolving situational pressures.

Accordingly, the analysis combined theory-informed exploration with openness to themes emerging from participants’ accounts. While the HBM helped identify key cognitive-perceptual factors underlying disclosure decisions, analytic flexibility was maintained to capture experiences that were not fully encompassed by the original model but were highly relevant to explaining disclosure decision-making in context. These included the emotional shock experienced after diagnosis, different disclosure pathway patterns, and post-disclosure outcomes and support needs. This approach enabled a more contextually grounded understanding of how newly diagnosed PLWH considered whether, when, and how to disclose their HIV status to sexual partners.

Data were collected through semi-structured in-depth interviews focusing on participants’ experiences, perceptions, and decision-making processes regarding sexual partner disclosure. The initial interview guide was developed on the basis of a literature review and the core constructs of the HBM, and was finalized after discussion and revision by three experts in relevant fields (Supplementary material). This study was reported in accordance with the Consolidated Criteria for Reporting Qualitative Research (COREQ).

### 2.3 Data collection

Data were collected through one-to-one semi-structured in-depth interviews. The interviews were conducted by researchers who had received standardized qualitative research training, and the interviewers had no direct therapeutic relationship with the participants. Before each interview, the participants were informed of the study purpose, interview content, principles of confidentiality, and audio-recording arrangements. A brief conversation was also conducted to establish a trusting relationship and facilitate the full expression of their real experiences. The interviews were conducted in a quiet, private, and independent setting. Written informed consent was obtained before the interview, and the whole interview process was audio-recorded with the participants’ permission. During the interviews, techniques such as open-ended questioning, probing, and clarification were used to guide the participants to provide in-depth accounts of their emotional experiences after diagnosis, decision-making regarding disclosure to sexual partners, influencing factors, and support needs. Each interview lasted approximately 30–60 min, and supplementary interviews were arranged when necessary. Non-verbal information and field observations were recorded simultaneously during the interviews to form interview memos. Audio recordings were transcribed within 24 h after each interview and checked against the original recordings. All data were anonymized by replacing real names with codes, such as P1, P2, and P3.

### 2.4 Data analysis

Data were analyzed using directed content analysis with support from NVivo 20 (Lumivero, United States). The HBM informed the initial coding framework, particularly in relation to risk perception, perceived benefits, perceived barriers, cues to action, and self-efficacy. First, transcripts were read repeatedly to gain an overall understanding of the data. Second, meaning units relevant to decision-making regarding voluntary sexual partner disclosure were identified, condensed, and openly coded. Third, codes were compared and grouped into subcategories and broader themes. Data that could not be adequately captured by the original HBM constructs but recurred across interviews and showed independent explanatory value were retained and further developed inductively. Through cross-case comparison of disclosure outcomes, decision speed, degree of internal conflict, and perceived barriers, three recurring disclosure pathway patterns were identified: direct disclosure, delayed disclosure after internal deliberation, and non-disclosure. Coding and theme development were undertaken independently by two researchers, and disagreements were resolved through discussion, with a third researcher consulted when necessary.

### 2.5 Rigor

Before data collection, all researchers received standardized training in qualitative interviewing. A semi-structured interview guide was used to maintain consistency in core topics while allowing participants to elaborate on issues relevant to their own experiences. During the interviews and analysis, the research team engaged in reflexive discussion to reduce the influence of prior professional assumptions on questioning, coding, and interpretation. Because the interviews were conducted in an infectious disease medical center, the research team recognized the potential influence of professional power dynamics and social desirability bias. To minimize this influence, interviewers emphasized that there were no right or wrong answers, that participants’ disclosure decisions would not be judged, and that their responses would not affect their medical care. Interviewers also used neutral, non-directive questions and avoided expressing approval or disapproval of any disclosure choice. During analysis, transcripts, codes, and themes were repeatedly compared across researchers to enhance consistency and credibility. Team discussions were also used to examine whether the interpretations were adequately grounded in the data and to avoid mechanically forcing participants’ accounts into pre-existing HBM constructs.

### 2.6 Ethical considerations

This study was approved by the Ethics Committee of Chongqing Public Health Medical Center (approval No. 2026-029-02-KY). The study strictly followed the principles of informed consent, voluntary participation, the right to withdraw at any time, and privacy protection. Before the interviews, all participants were informed of the study purpose, content, potential risks and benefits, and all signed written informed consent forms. Audio recordings, transcripts, and related materials were kept by designated personnel and were used only for this study. When the study results are published, all findings will be presented anonymously, and no identifiable participant information will be disclosed.

## 3 Results

### 3.1 General information of the participants

A total of 31 newly diagnosed PLWH were interviewed in this study, and recruitment was stopped after data saturation was reached. Table 1 summarizes the general characteristics of the participants, illustrating participant heterogeneity and providing contextual information for interpreting the disclosure pathways.

**Table 1:** General characteristics of the participants.

Participant ID	Sex	Age	Education level	Partner type	Partner’s HIV status	Disclosure status
P1	Male	42	Junior high school	Wife	Positive	Disclosed
P2	Male	32	Junior college	Girlfriend	Positive	Disclosed
P3	Male	44	Junior college	Wife	Positive	Disclosed
P4	Male	53	Junior high school	Wife	Positive	Disclosed
P5	Male	56	Primary school	Wife	Not tested	Disclosed
P6	Female	26	Junior high school	Boyfriend	Positive	Disclosed
P7	Male	85	Junior college	Wife	Negative	Disclosed
P8	Male	77	Primary school	Wife	Not tested	Disclosed
P9	Male	34	Junior high school	Wife	Positive	Disclosed
P10	Male	27	Bachelor’s degree	Boyfriend	Positive	Disclosed
P11	Male	52	Primary school	Wife	Positive	Disclosed
P12	Male	52	Primary school	Wife	Positive	Disclosed
P13	Male	60	Junior high school	Wife	Not tested	Not disclosed
P14	Female	44	Junior college	Husband	Positive	Disclosed
P15	Female	54	Junior high school	Husband	Positive	Disclosed
P16	Male	70	Primary school	Wife	Positive	Disclosed
P17	Male	46	Primary school	Wife	Not tested	Not disclosed
P18	Male	50	Primary school	Wife	Negative	Not disclosed
P19	Male	49	Junior college	Boyfriend	Not tested	Not disclosed
P20	Male	40	Junior college	Wife	Not tested	Disclosed
P21	Male	75	Primary school	Wife	Not tested	Disclosed
P22	Female	65	Primary school	Husband	Not tested	Not disclosed
P23	Male	53	Primary school	Wife	Positive	Disclosed
P24	Male	61	Primary school	Wife	Positive	Disclosed
P25	Male	47	Junior high school	Wife	Positive	Disclosed
P26	Male	57	Junior college	Wife	Negative	Disclosed
P27	Male	63	Junior high school	Wife	Not tested	Disclosed
P28	Male	41	Bachelor’s degree	Wife	Negative	Disclosed
P29	Male	70	Primary school	Wife	Positive	Disclosed
P30	Male	69	Junior high school	Wife	Not tested	Disclosed
P31	Male	68	Primary school	Wife	Positive	Disclosed

This table was used to describe participant heterogeneity and provide contextual information for interpreting the disclosure pathways.

### 3.2 Results of theme extraction

Based on the analysis of the interview data, and focusing on the decision-making process of voluntary disclosure to sexual partners among newly diagnosed PLWH, six themes and 18 subthemes were ultimately identified, namely emotional shock, risk perception, perceived benefits, barriers, disclosure pathways, and outcomes and support (Table 2).

**Table 2:** Summary of themes and subthemes.

Theme	Subthemes
Emotional shock	(1) Emotional shock and crisis experience; (2) Stigma and practical pressure; (3) Rational acceptance and adaptation
Risk perception	(1) Clear risk awareness; (2) Contextualized risk judgment; (3) Insufficient risk perception
Perceived benefits	(1) Health protection; (2) Relational and psychological benefits; (3) Insufficient perception of benefits
Barriers	(1) Social and relational risks; (2) Concerns about privacy control; (3) Insufficient knowledge and communication
Disclosure pathways	(1) Direct disclosure; (2) Delayed disclosure after internal deliberation; (3) Non-disclosure
Outcomes and support	(1) Relational outcomes after disclosure; (2) Differences in self-efficacy; (3) Structure of support needs

#### 3.2.1 Emotional shock

After learning their diagnosis, newly diagnosed PLWH generally experienced emotional reactions to varying degrees, which were mainly reflected in three aspects: emotional shock and crisis experience, stigma and practical pressure, and rational acceptance and adaptation.

##### 3.2.1.1 Emotional shock and crisis experience

Some participants first showed obvious emotional shock after diagnosis, mainly including shock, fear, anxiety, and a strong sense of crisis, and a few individuals even experienced despair.

*One participant said, “It felt as if the sky had fallen. I felt that life had lost its meaning after getting this disease… I even thought about dying” (P6).*

*Another participant mentioned, “I really could not believe it. I had always been careful about these things, so I could not understand how I had suddenly become infected” (P25).*

*Another participant said, “My heart sank at that moment. I was stunned. I never thought it would be this disease” (P28).*

*For some participants, emotional shock was also accompanied by obvious physical discomfort, such as fever, fatigue, dizziness, and sleep disturbance. A few also showed tendencies toward avoidance or denial, such as being “reluctant to think too much about this result” (P28).*

This suggests that, for some PLWH, the newly diagnosed stage is a highly vulnerable period of psychological crisis.

##### 3.2.1.2 Stigma and practical pressure

In addition to the shock brought by the disease itself, feelings of stigma and pressures in real life were also important components of the participants’ emotional experiences. Some participants showed obvious shame and concern about social evaluation after diagnosis.

*One participant mentioned, “I felt that this disease was a stain, and I was ashamed to let others know. If other people found out, they would definitely look down on me and reject me” (P18).*

*Another participant said, “I didn’t dare tell people around me, because I was afraid that if they knew, they would have a bad impression of me” (P24).*

At the same time, some middle-aged and older participants placed greater emphasis on economic pressure and family responsibilities.

*One participant said, “What worries me most is the financial burden… I’m afraid of becoming a burden to my family; I’m also worried that no one will take care of the older adult(s), and that my child’s life will be affected” (P31).*

*Another participant mentioned, “Treatment costs money, and life at home is already tight. Where would the money for treatment come from?” (P18).*

These findings suggest that the psychological burden after diagnosis stemmed not only from the disease itself, but also from multiple practical pressures, including economic strain, family responsibilities, and social stigma.

##### 3.2.1.3 Rational acceptance and adaptation

Some participants also showed relatively stable emotional states after diagnosis, were able to accept reality more quickly, and shifted to rational coping.

*One participant said, “I just treat it as normally as an ordinary chronic disease” (P27).*

*Another participant mentioned, “I didn’t have any special worries, and I didn’t feel uncomfortable inside” (P21).*

*In addition, although some participants experienced brief emotional fluctuations in the early stage after diagnosis, they soon showed a strong attitude of accepting reality, as reflected in statements such as “Now that it has happened, I can only face it” (P31) and “Once I knew I had the disease, I had to face it” (P24).*

This indicates that some newly diagnosed PLWH were able to complete psychological adjustment relatively quickly and gradually develop the understanding that the disease is treatable, manageable, and something that needs to be faced.

#### 3.2.2 Risk perception

After diagnosis, participants generally assessed the potential risk of HIV infection faced by their sexual partners, but there were clear differences in their levels of understanding and ways of making judgments. These differences were mainly reflected in three situations: clear risk awareness, contextualized risk judgment, and insufficient risk perception.

##### 3.2.2.1 Clear risk awareness

Most participants were able to recognize after diagnosis that their sexual partners might face a potential risk of infection, and regarded their partners’ health as an important consideration.

*One participant said, “As soon as I was diagnosed, I asked her to get tested immediately. When the result came back negative, I finally felt relieved” (P28).*

*Another participant mentioned, “If I don’t tell her, she won’t go for testing, and if there is any risk, it will be delayed” (P20).*

*Some participants directly viewed disclosure as an important measure to prevent transmission, such as, “She has to know, so that I can avoid passing it on to her” (P11).*

These statements suggest that awareness of partner-related risk is an important prerequisite for the intention to disclose, but it does not necessarily translate into actual disclosure behavior.

##### 3.2.2.2 Contextualized risk judgment

Some participants did not make judgments based on general knowledge of HIV transmission, but instead assessed risk in light of their own life circumstances.

*One participant believed, “We haven’t had sex for quite a while, so I think her health risk is not that high” (P27).*

*Another participant said, “We’ve been sleeping separately for years and haven’t had contact, so it probably wouldn’t be transmitted” (P29).*

*Another participant mentioned, “I’ve always been very careful, and neither of us had anyone else, so the risk was mainly during that earlier period” (P28).*

These judgments, to some extent, reflect the participants’ basic understanding of transmission routes, but their risk assessment relied more on personal experience than on systematic medical knowledge.

##### 3.2.2.3 Insufficient risk perception

Some participants still lacked a clear understanding of HIV transmission routes and infection risk, which was reflected in vague or underestimated judgments of risk.

*One participant mentioned, “I really don’t understand the routes of transmission at all. I don’t know anything about it” (P29).*

*Another participant said, “I can’t really say how great the risk is” (P18).*

*Another participant believed that “as long as there is no contact in daily life, nothing will happen,” but was unable to clearly distinguish specific routes of transmission (P12).*

In addition, although a few participants were aware that risk existed, they did not further consider the possible consequences of non-disclosure, showing a certain degree of insufficient risk perception or delay in decision-making.

#### 3.2.3 Perceived benefits

In the decision-making process regarding whether to voluntarily disclose HIV infection status to sexual partners, participants differed in their understanding of the benefits of disclosure. These differences were mainly reflected in three aspects: health protection, relational and psychological benefits, and insufficient perception of benefits.

##### 3.2.3.1 Health protection

For most participants, the most direct and central benefit of disclosure was the protection of their sexual partners’ health by prompting them to undergo testing as early as possible, take timely protective measures, or receive treatment.

*One participant stated, “The most important thing was to let my wife know as soon as possible and get tested right away to check the risk of infection. This was for her own good, and it was also what I should do as a family member” (P25).*

*Another participant mentioned, “The benefits are very obvious. First, it allows her to know the situation in time, go for screening with peace of mind, and protect her health” (P28).*

*Another participant believed that disclosure could prevent a partner from continuing to be exposed to the risk of infection without knowing it: “If I don’t tell her, she won’t go for testing, and that should affect her health” (P11).*

These findings indicate that, among most participants who had completed disclosure, health protection constituted the core content of perceived benefits.

##### 3.2.3.2 Relational and psychological benefits

In addition to health protection, some participants also understood disclosure as an important way to maintain honesty in the relationship, obtain support from their partners, and reduce their own psychological burden.

*One participant said, “The benefit is that I don’t need to hide it, and I don’t feel burdened inside. We can be honest with each other” (P27).*

*Another participant also mentioned, “Second, it removed the burden of hiding it in my heart. Once the two of us were honest with each other, the weight on my mind was lifted, and I actually felt more at ease getting along with her” (P28).*

*Another participant believed that after disclosure, “we could face the treatment together—do the tests that need to be done and receive the treatment that needs to be received. I can’t carry it all by myself” (P31).*

Therefore, relational and psychological benefits were also important motivations for some participants to make the decision to disclose.

##### 3.2.3.3 Insufficient perception of benefits

A small number of participants lacked a clear understanding of the benefits of disclosure, and some even explicitly stated that they “could not see any benefits” or believed that “disclosure was meaningless.”

*One participant stated directly, “I really do not see any benefit at all, not even one” (P22).*

*Another participant said, “For now, I haven’t thought about any benefits of telling her. All I can see are the conflicts that would arise after disclosure, and I haven’t considered the positive side” (P18).*

*Another participant mentioned, “There’s no benefit in telling her. Even if I tell her, it won’t make any difference” (P13).*

These findings suggest that, among some participants who chose not to disclose or hesitated to disclose, the perception of benefits had not been fully established, or had been overshadowed by concerns about relationship conflict, privacy disclosure, and social stigma.

#### 3.2.4 Barriers

Although most participants ultimately disclosed their HIV status to their sexual partners, many still mentioned barriers of varying degrees during the decision-making process. These barriers were mainly concentrated in three aspects: social and relational risks, concerns about privacy control, and insufficient knowledge and communication.

##### 3.2.4.1 Social and relational risks

For some participants, the greatest barrier to disclosure was not the disease itself, but the possible changes in their relationship and the social consequences that might be triggered after disclosure.

*One participant said, “The biggest difficulty is psychological. I’m afraid she will reject me, and I’m afraid it will affect the relationship we’ve had for so many years” (P28).*

*Another participant mentioned, “If I tell her, it will definitely lead to arguments. Our relationship of so many years may break down, and the family will also be full of quarrels every day” (P18).*

*Another participant repeatedly struggled before disclosure and felt that “on the one hand, I thought I should tell her so that she could protect herself, but on the other hand, I was afraid that if I told her, I would be disliked and isolated. I felt extremely conflicted” (P4).*

These findings indicate that relational risks and social risks were often intertwined and together constituted important psychological barriers to disclosure.

##### 3.2.4.2 Concerns about privacy control

Among some participants who had not disclosed or tended not to disclose, concerns about privacy control were particularly prominent.

*One participant stated clearly, “The biggest barrier is that he cannot keep things confidential. If I tell him, it would be almost the same as telling all the relatives” (P22).*

*Another participant mentioned, “The more people who know, the worse it will be if rumors spread later. I’m afraid others will spread it around, and that it will affect me badly” (P13).*

*Another participant also said that they “did not want more family members to know,” and therefore tended not to disclose from the very beginning (P22).*

This suggests that, for some participants, the issue of whether to disclose was not only a matter of “whether to say it,” but also of “whether it could still be controlled after being said.”

##### 3.2.4.3 Insufficient knowledge and communication

Insufficient knowledge and lack of confidence in communication were also important barriers faced by some participants during the disclosure process.

*One participant said, “I really don’t understand the routes of transmission or the treatment methods. I don’t know anything, so I feel a bit uncertain” (P29).*

*Another participant mentioned, “At that time, I hadn’t fully accepted the disease myself, and I didn’t know how to start the conversation. This kind of struggle and hesitation lasted for quite a while” (P28).*

*Another participant hoped that healthcare professionals could “guide patients on how to start the conversation with their partners and provide some communication skills and wording” (P27).*

These findings suggest that insufficient knowledge and communication do not necessarily lead to non-disclosure, but they can increase hesitation about disclosure and psychological burden.

#### 3.2.5 Disclosure pathways

A comprehensive analysis of the interview data showed that voluntary disclosure to sexual partners among newly diagnosed PLWH did not follow a single pathway, but rather showed clear differentiation. According to the speed of decision-making, degree of internal conflict, and final behavioral outcomes, three main pathways could be summarized: direct disclosure, delayed disclosure after internal deliberation, and non-disclosure. Among the 31 participants, 26 had disclosed their HIV status to their sexual partners and 5 had not disclosed. Among those who disclosed, 21 participants described direct disclosure, whereas 5 participants described delayed disclosure after internal deliberation. The remaining 5 participants were categorized into the non-disclosure pathway. To further illustrate the differences among the three disclosure pathways, Table 3 summarizes their main characteristics in terms of time duration to disclosure decision, internal struggle or hesitancy, perceived barriers, and disclosure outcome.

**Table 3:** Type of disclosure pathways among newly diagnosed PLWH.

Disclosure pathway	Time duration to disclosure decision	Internal struggle/Hesitancy	Perceived barriers	Outcome
Direct disclosure	Fast, usually soon after diagnosis	None or minimal	Low or manageable	Disclosed
Delayed disclosure after internal deliberation	Delayed, after a period of hesitation and psychological conflict	High but eventually resolved	Moderate to high, but eventually overcome	Disclosed
Non-disclosure	Indefinite delay or no clear disclosure plan	High or unresolved, or a firm decision not to disclose	Very high, especially privacy concerns, stigma, and anticipated relationship conflict	Not disclosed

##### 3.2.5.1 Direct disclosure

Direct disclosure was the most common pathway in this study. Participants in this group made the decision to disclose relatively quickly after diagnosis, usually without showing obvious internal struggle and with less perception of strong barriers.

*One participant said, “There was absolutely no struggle at all! I told him the same day I got the test result. I didn’t hesitate at all, and I was very sure that he should know as soon as possible” (P15).*

*Another participant also mentioned, “There were no difficulties or barriers at all, and I never struggled. I think this kind of thing should just be said directly. There’s no need to hide it” (P27).*

*Another participant “called his wife and told her immediately after the diagnosis, without any delay at all,” believing that “this kind of thing simply cannot be concealed” (P25).*

##### 3.2.5.2 Delayed disclosure after internal deliberation

Participants who delayed disclosure after internal deliberation experienced evident psychological conflict regarding whether to disclose their HIV status.

*One participant said, “Yes, I struggled. Deep down I knew I had to tell her. It was being responsible for her and also for our relationship. But on the other hand, I was very afraid and worried that she would react intensely” (P28).*

*Another participant also mentioned, “During those 7 days, I couldn’t sleep at all. On the one hand, I felt I should tell her so that she could protect herself, but on the other hand, I was afraid that if I told her, I would be disliked and isolated. I felt extremely conflicted” (P4).*

*Another participant hesitated briefly between whether to tell or not because the emotional foundation of the relationship was only average and there were concerns about the impact on the children, but eventually “still gathered the courage to tell” (P14).*

These findings suggest that, although such participants experienced obvious struggle, they eventually completed disclosure under the combined influence of a sense of responsibility, concern about possible consequences, and external support. For these participants, the eventual decision to disclose was often facilitated by specific cues to action, such as heightened concern about the partner’s need for testing, repeated consideration of possible transmission risk, and encouragement or guidance from healthcare professionals or family members. These cues helped transform perceived responsibility into actual disclosure behavior after a period of hesitation and internal deliberation.

##### 3.2.5.3 Non-disclosure

Participants in the non-disclosure group clearly chose not to disclose, or chose not to disclose for the time being, and formed a relatively stable decision-making position.

*One participant stated clearly, “No, not at all. I did not struggle with it. I was very firm from the beginning that I would not tell her. There was nothing for me to hesitate about” (P18).*

*Another participant also mentioned, “I didn’t struggle at all. From the very beginning, I was inclined not to tell him. I never even thought about telling him about the disease. I was very sure that I would not disclose it” (P22).*

*Another participant believed that “there’s no benefit at all—why tell her? Telling her would only make things worse, and it’s useless anyway,” and adopted an alternative way to deal with the risk by “asking his daughter to call his wife in for testing under the name of another illness” (P13).*

This reflected an indirect risk-management strategy, in which the participant attempted to reduce the partner’s health risk without directly disclosing his HIV status.

This pathway was usually associated with higher perceived barriers, especially concerns about privacy, anticipated relationship conflict, and worries about loss of control over information.

#### 3.2.6 Outcomes and support

After disclosure had been completed or not completed, participants showed different characteristics in terms of relationship changes, self-efficacy, and support needs. These outcomes further reflected the differences in their disclosure decision-making pathways.

##### 3.2.6.1 Relational outcomes after disclosure

Overall, most participants who had completed disclosure did not experience the severe negative consequences they had anticipated. Instead, to a certain extent, they obtained understanding and support from their partners.

*One participant said, “After I told her, although she was shocked at first, she calmed down quickly and accompanied me for the follow-up examinations. She didn’t make a scene, and that really reassured me” (P28).*

*Another participant also mentioned, “After my spouse knew, she didn’t reject me, and that was enough for me” (P29).*

*Another participant believed that “our relationship was already good, and disclosure would not affect our relationship with each other” (P27).*

These findings suggest that, for most individuals who completed disclosure, the actual relational outcomes were often better than they had expected beforehand.

##### 3.2.6.2 Differences in self-efficacy

Participants from different disclosure pathways showed clear differences in self-efficacy. Most participants who had completed disclosure demonstrated relatively high self-efficacy.

*One participant said, “I have the ability to cope. I don’t think there will be any serious consequences. We are all adults and can face it rationally” (P27).*

*Another participant mentioned, “After seeing her reaction, I instead felt that I had the ability to cope” (P28).*

*Another participant believed that “even if we separated, I could accept it” (P1).*

*In contrast, among participants who had not disclosed or tended not to disclose, coping was more often manifested as avoidance and information control, such as “as long as I don’t say it, those troubles won’t happen” (P22).*

##### 3.2.6.3 Structure of support needs

In terms of support needs, participants’ expressions were mainly concentrated in three aspects: disease-related knowledge, psychological support, and practical resources.

*Some participants stated that they had insufficient understanding of transmission routes, treatment methods, and protective measures, and hoped that healthcare professionals could “clearly explain how it can be treated and how to protect against it” (P27).*

*Some participants also raised the need for psychological support and hoped that “there could be a place to hear about other people’s experiences,” so as to reduce feelings of loneliness and uncertainty (P28).*

*In addition, some middle-aged and older participants, or those with heavier family burdens, placed greater emphasis on the need for financial assistance or policy support, such as “what worries me most is still the financial problem” (P31).*

*At the level of disclosure decision-making, some participants suggested that healthcare professionals should provide more specific communication guidance, such as “teaching patients how to start the conversation” and “allowing partners to receive counseling together when necessary” (P28).*

Overall, the support needs expressed by participants showed a structural pattern in which knowledge support served as the foundation, while psychological support and practical support jointly promoted disclosure decision-making and subsequent coping.

## 4 Discussion

### 4.1 Dynamic decision-making characteristics of disclosure to sexual partners during the newly diagnosed stage

This study showed that voluntary disclosure to sexual partners among newly diagnosed PLWH was not a one-time or purely rational behavioral choice, but a dynamic decision-making process shaped by emotional shock after diagnosis, perceptions of partner-related risk, anticipated benefits of disclosure, and concerns about potential negative consequences. Participants often moved from an initial period of emotional crisis to a stage of active weighing of whether to disclose, when to disclose, and how to disclose. This suggests that disclosure decision-making in the newly diagnosed stage is both emotionally embedded and context-dependent, rather than simply determined by knowledge of HIV transmission or a sense of moral responsibility alone (5, 6, 15).

Our findings are broadly consistent with previous studies showing that HIV disclosure is not a simple binary outcome of disclosure versus non-disclosure, but a gradual and adaptive process shaped by stress, coping, and interpersonal context (5, 15). Compared with previous studies, the present study further highlights the particularity of the newly diagnosed stage. In this early period, participants were still coping with emotional shock, uncertainty, and stigma-related concerns, which often delayed their ability to engage in calm and rational disclosure planning. This is consistent with earlier evidence suggesting that the period immediately following diagnosis is a psychologically vulnerable stage and may require timely mental health and counseling support (6).

At the same time, the motivations and obstacles described by participants were not static. Perceived responsibility for protecting partners’ health, the wish to maintain honesty in intimate relationships, and the need to reduce psychological burden could all promote disclosure, whereas anticipated stigma, fear of relationship disruption, privacy concerns, and low confidence in communication could inhibit it. These findings are generally in line with previous studies on facilitators and barriers to HIV partner disclosure (7–9, 11–14). Overall, the findings indicate that voluntary disclosure during the newly diagnosed stage is best understood as a dynamic behavioral process emerging from the interaction between emotional responses, cognitive appraisal, and contextual pressures. This also suggests that post-diagnosis services should move disclosure-related support to an earlier stage and provide not only information, but also emotional reassurance, communication guidance, and privacy-sensitive support (4, 6, 18).

### 4.2 The formation process of voluntary disclosure decisions to sexual partners under the HBM framework

Guided by the Health Belief Model (HBM), this study found that voluntary disclosure to sexual partners among newly diagnosed PLWH was shaped by the combined influence of perceived susceptibility and severity, perceived benefits, perceived barriers, cues to action, and self-efficacy, rather than by any single factor alone. In this sense, disclosure was not simply a moral or interpersonal choice, but a health-related behavioral decision developed through ongoing cognitive and emotional appraisal (16, 17). Participants were more likely to consider disclosure when they recognized the possible risk of HIV transmission to their partners, anticipated meaningful benefits from disclosure, and felt able to cope with the consequences. By contrast, when anticipated barriers such as stigma, relational conflict, privacy loss, or communication difficulty outweighed perceived benefits, delayed disclosure or non-disclosure became more likely.

First, perceived susceptibility and perceived severity constituted an important cognitive foundation for the initiation of disclosure behavior. When individuals clearly recognized that their partners were at potential risk of infection and realized that non-disclosure might delay testing, protection, or treatment, they were more likely to form an intention to disclose (7–9). Second, perceived benefits were an important factor driving the formation of disclosure behavior. This study showed that the benefits of disclosure were reflected not only in protecting the partner’s health, but also in maintaining honesty in the relationship, obtaining support from the partner, and reducing psychological burden (18, 19). In contrast, perceived barriers were the key mechanism inhibiting disclosure behavior, mainly manifested as concerns about stigma and discrimination, the risk of relationship breakdown, worries about privacy disclosure, and insufficient confidence in communication. Especially during the newly diagnosed stage, when individuals have not yet completed psychological adjustment, they are more likely to amplify the possible negative consequences of disclosure (6, 8, 14). In addition, cues to action played an important triggering role in disclosure decision-making. Advice from healthcare professionals, the partner’s need for testing, and concerns about transmission risk could all prompt individuals to move from cognitive awareness to actual action (4, 18). Self-efficacy, in turn, determined whether individuals could translate their intention to disclose into actual behavior. Those who had completed disclosure usually had stronger confidence in communication and greater ability to bear the consequences, whereas those who had not disclosed were more likely to reduce risk by avoiding discussion or controlling the spread of information (16, 17).

It should also be noted that, although the HBM provided a useful explanatory framework for understanding the core psychological mechanisms involved in disclosure decision-making, not all themes identified in this study could be fully contained within the original model. Themes such as emotional shock, disclosure pathways, and outcomes and support more strongly reflected the contextual and processual features of the newly diagnosed stage. Rather than weakening the relevance of the HBM, these findings extend its application by showing how model-related perceptions are activated, interrupted, strengthened, or reshaped in real-life contexts (5, 6, 14). Overall, the present findings suggest that newly diagnosed PLWH are more likely to complete voluntary partner disclosure when partner-related risk is clearly recognized, expected benefits are meaningful, perceived barriers are manageable, and sufficient support and coping confidence are available. Conversely, when barriers dominate and self-efficacy remains low, postponement or non-disclosure becomes more likely (16, 17).

### 4.3 Differences in disclosure pathways reflect the differentiation of behavioral patterns

This study identified three recurring disclosure pathway patterns among newly diagnosed PLWH: direct disclosure, delayed disclosure after internal deliberation, and non-disclosure. This finding is important because it shows that voluntary disclosure to sexual partners should not be understood as a simple binary outcome. Instead, disclosure behavior appears to develop through different trajectories shaped by emotional state, perceived partner-related risk, anticipated benefits, perceived barriers, and coping confidence. In this sense, the decision to disclose is not merely a matter of willingness, but a differentiated behavioral outcome formed under the combined influence of multiple psychological and contextual factors (16, 17).

Participants in the direct disclosure pathway tended to show clearer awareness of partner-related health risk, a stronger sense of interpersonal responsibility, and fewer perceived barriers to disclosure. For these individuals, disclosure was often regarded as an immediate and necessary response, which is broadly consistent with previous studies showing that concern for partner protection and relational responsibility can facilitate disclosure (7–9). By contrast, those in the delayed disclosure after internal deliberation pathway more clearly reflected the complexity of the newly diagnosed stage. Although they recognized the importance of disclosure, they simultaneously worried about rejection, relationship disruption, emotional confrontation, or loss of privacy, and therefore experienced substantial psychological conflict before eventually disclosing. This pattern is also in line with prior qualitative evidence indicating that newly diagnosed individuals may undergo prolonged internal negotiation before disclosure (4–6, 14).

Participants in the non-disclosure pathway were more likely to perceive disclosure as a high-risk act whose possible negative consequences outweighed its expected benefits. In this group, privacy concerns, fear of stigma, and anticipated relationship damage were especially prominent, and some participants preferred indirect or avoidant ways of managing risk rather than direct disclosure (20, 21). Rather than replacing the HBM, these three pathways can be understood as different processual expressions of how HBM-related perceptions and contextual pressures are translated into behavior during the newly diagnosed stage (16, 17). These differentiated pathways also suggest the need for pathway-sensitive support in practice: individuals who are ready for direct disclosure may benefit most from immediate partner-testing referral and practical counseling; those who are struggling may require emotional support, guided communication strategies, and reassurance around disclosure consequences; and those who are not ready to disclose may need more privacy-sensitive counseling, stigma reduction support, and alternative risk-management discussions before disclosure can be realistically considered (18, 22).

### 4.4 Implications for clinical practice and partner services

These findings have implications not only for individual post-diagnosis counseling, but also for the design of HIV partner services and early support pathways. First, assessment of partner-related disclosure needs should be incorporated into routine counseling soon after diagnosis, as this period appears to be a critical window during which disclosure intentions and behavioral pathways begin to form. Rather than waiting until patients raise the issue themselves, healthcare providers may proactively explore whether the patient has a current sexual partner, what concerns they hold about disclosure, and what kind of support they may need (4, 6, 18).

Second, support for partner disclosure should move beyond the provision of disease-related knowledge alone. Although knowledge about transmission risk, testing, and protection is essential, this study suggests that emotional reassurance, communication coaching, and confidence-building are equally important during the newly diagnosed stage. For many participants, the difficulty of disclosure lay not only in understanding HIV, but also in managing fear, anticipating interpersonal consequences, and finding an acceptable way to start the conversation. Therefore, disclosure-related interventions should shift from simple information delivery to multidimensional support that combines knowledge, emotion, and communication (18, 23).

Third, privacy protection should be treated as a central component of partner services rather than a peripheral concern. Some participants were not opposed to disclosure in principle, but were deeply concerned about losing control over who else might learn their HIV status after telling a partner. This suggests that counseling should explicitly address confidentiality boundaries, possible disclosure scenarios, and available support options, so as to improve trust in healthcare services and reduce disclosure-related fear (8, 9).

Finally, the findings support a more stratified and pathway-sensitive approach to service delivery. Individuals in different disclosure pathways may require different forms of support, ranging from immediate referral for partner testing to staged emotional counseling and privacy-sensitive communication planning. Beyond individual clinical encounters, these findings may help inform the development of structured partner-service pathways, early support protocols, and communication training for frontline HIV care providers (18, 24).

In the local public health context of Chongqing, voluntary partner disclosure should also be understood in relation to partner notification and partner testing services. Although participants’ disclosure choices generally aligned with the public health goal of facilitating timely partner testing and reducing secondary transmission, these choices were also shaped by concerns about stigma, privacy control, and relationship consequences (25). Therefore, partner services should emphasize voluntary participation, confidentiality protection, individualized counseling, and linkage to partner testing, so as to balance public health needs with respect for individual autonomy and privacy.

### 4.5 Limitations

This study has several limitations. First, this study adopted a descriptive qualitative design, and the sample was drawn from one tertiary infectious disease specialty hospital in Chongqing. Therefore, the findings should be interpreted within this specific institutional and regional context, and may not fully represent the experiences of newly diagnosed PLWH in other regions, community-based settings, or different levels of healthcare institutions. Second, this study was mainly based on participants’ self-reported data, and disclosure behaviors and their consequences may have been affected by recall bias or social desirability bias. In particular, when sensitive issues such as stigma, relationship conflict, and privacy management were involved, some participants may have withheld their true thoughts or experiences. Third, this study focused on participants’ subjective experiences and decision-making logic during the newly diagnosed stage, and did not further incorporate the perspectives of sexual partners or healthcare professionals, nor did it include longitudinal follow-up to examine how disclosure decisions and relationship outcomes evolved over time. In addition, although the HBM provided a useful guiding framework for identifying key mechanisms influencing disclosure behavior, it may also have influenced the open interpretation of the data to some extent. Future multicenter qualitative or mixed-methods studies involving more diverse settings, longitudinal follow-up, and the perspectives of partners and healthcare professionals are needed to further clarify how disclosure decision-making evolves across relationship and service contexts.

## 5 Conclusion

Voluntary disclosure to sexual partners among newly diagnosed PLWH is not a single, static behavioral choice, but a dynamic decision-making process formed under the combined influence of emotional shock after diagnosis, risk appraisal, perceived benefits, and the weighing of barriers. Based on the Health Belief Model, this study found that awareness of partner-related health risks and expectations of the benefits of disclosure were important foundations driving disclosure behavior, whereas concerns about stigma, relational risks, worries about loss of privacy control, and insufficient confidence in communication were key factors delaying or hindering disclosure. Different individuals may present three pathways: direct disclosure, delayed disclosure after internal deliberation, and non-disclosure. These findings suggest that post-diagnosis HIV services should introduce disclosure support earlier and combine knowledge provision with psychological counseling, communication guidance, privacy protection, and pathway-sensitive support.

## Data Availability

The original contributions presented in the study are included in the article/Supplementary material, further inquiries can be directed to the corresponding author.
