# Supplementary material for: Decision-making pathways of voluntary sexual partner disclosure among newly diagnosed people living with HIV: a qualitative study informed by the health belief model
Source: Front Public Health. 2026 Jun 8;14:1863157. doi: 10.3389/fpubh.2026.1863157 (PMC13284106; doi:10.3389/fpubh.2026.1863157)
Supplement: Supplementary file 1 [file Data_Sheet_1.DOCX]

**Supplementary Interview Guide**Qualitative interview guide for exploring the decision-making process of voluntary sexual partner disclosure among newly diagnosed people living with HIV, informed by the Health Belief Model

1. Initial reactions after a new HIV diagnosis – Perceived severity

(1) After learning that you had been diagnosed with HIV, what was your first reaction and what were your main feelings at that time?
Probing questions: What worried you most? Was there a period during which you intentionally avoided thinking about it?

2. Assessment of transmission risk and consequences – Perceived susceptibility
(2) After the diagnosis, did you consider the possible health risks faced by your sexual partner? How did you judge that risk?
(3) If you did not disclose your HIV status to your sexual partner, what consequences did you think might follow? If you did disclose, what consequences did you think might occur?

3. Internal conflict and weighing of considerations – Perceived benefits and perceived barriers
(4) In your view, what possible benefits might come from disclosing your HIV status to your sexual partner?
(5) When considering whether to disclose your HIV status to your sexual partner, what concerns, difficulties, or barriers did you experience?
(6) During this period, did you ever find yourself repeatedly struggling between “I should tell” and “I do not want to tell”? Could you describe that experience?

4. Cues to action and triggering factors – Cues to action
(7) Before you made your decision, were there any events or suggestions from other people that had an important influence on you?
(8) Was there any key event or moment that changed your thinking about whether to disclose your HIV status to your sexual partner?

5. Disclosure outcomes and coping capacity – Self-efficacy
(9) At present, have you disclosed your HIV status to your sexual partner? If not, how did you make that decision, and why?
(10)When thinking about disclosure to your sexual partner, did you feel that you had the ability to cope with the possible consequences?

6. Support needs and recommendations – Service support and intervention suggestions
(11) In the process of deciding whether to disclose your HIV status to your sexual partner, what kinds of support did you consider most important? What support is currently lacking?
(12) In your opinion, how could healthcare professionals or public health workers better support newly diagnosed people living with HIV in facing disclosure decisions?
